# Use of Yarn and Carded Jute as Epoxy Matrix Reinforcement for the Production of Composite Materials for Application in the Wind Sector: A Preliminary Analysis for the Manufacture of Blades for Low-Intensity Winds

**DOI:** 10.3390/polym15183682

**Published:** 2023-09-07

**Authors:** Robson Luis Baleeiro Cardoso, Jean da Silva Rodrigues, Roberto Paulo Barbosa Ramos, Alessandro de Castro Correa, Elza Monteiro Leão Filha, Sergio Neves Monteiro, Alisson Clay Rios da Silva, Roberto Tetsuo Fujiyama, Verônica Scarpini Candido

**Affiliations:** 1 Engineering of Natural Resources of the Amazon Program, Federal University of Para—UFPA, Belem 66075-110, Brazil; rlbaleeiro@gmail.com (R.L.B.C.); fujiyama@ufpa.br (R.T.F.); 2 Materials Engineering Program, Federal Institute of Education, Science and Technology of Para—IFPA, Belem 66645-240, Brazil; jean.rodrigues@ifpa.edu.br (J.d.S.R.); roberto.ramos@ifpa.edu.br (R.P.B.R.); alessandro.correa@ifpa.edu.br (A.d.C.C.); elza.filha@ifpa.edu.br (E.M.L.F.); 3 Materials Science Program, Military Engineering Institute—IME, Rio de Janeiro 22290-270, Brazil; snevesmonteiro@gmail.com; 4 Material Science and Engineering Program, Federal University of Para—UFPA, Ananindeua 67000-000, Brazil; alissonrios@ufpa.br

**Keywords:** natural fiber, carded jute, composite material, ANOVA

## Abstract

The development of wind turbines for regions with low wind speeds imposes a challenge to the expansion of the corresponding energy generation capacity. The present work consists of an evaluation of the potential carded jute fiber and jute yarn to be used in the construction of a wind blade for regions of low wind intensity. The fibers used were supplied by Company Textile of Castanhal (Castanhal-Para-Brazil) and used in the study without chemical treatment in the form of single-filament fibers and yarns with a surface twist of 18.5°. The composites were produced through the resin infusion technique and underwent tensile and shear tests using 120-Ohm strain gauges and a blade extensometer to obtain the Young’s modulus. In the analysis of the results, the ANOVA test was applied with a 0.05 significance level, followed by Tukey’s test. The results showed that long, aligned jute fibers can be a good option for laminated structures applied in composites for small wind turbine blades.

## 1. Introduction

The ever-increasing demand for electricity has fueled the expansion of the global wind energy industry. Wind energy is one of the most promising power generation technologies [1]. However, areas with high wind intensity are becoming increasingly scarce as more wind farms are installed each year [2], which makes small turbine designs developed for low-wind-speed areas particularly attractive.

In wind power generation, the synthetic materials commonly used in the components are glass and carbon fiber, which are not environmentally friendly [3]. Indeed, they come from costly and energy-intensive manufacturing processes that have economic and environmental impacts [4]. In addition, the disposal of synthetic fibers, or the materials from which they are made, has been a challenge [5,6]. Consequently, to make wind power generation more environmentally friendly, the introduction of natural lignocellulosic fibers (NLFs) in composite structures [7] can be an alternative in the search to reduce the industrial cost, the process stages, the discarding, and the pollution of the environment [8,9].

NLFs have comparable mechanical properties to and are less dense than synthetic fibers, such as glass fiber [10], which is widely used in the wind energy industry. Moreover, the use of the natural fibers adds social and economic value, reduces industrial steps, reduces spending on electricity, and minimizes costs [10,11], playing an important role in polymer composites [5,12].

**Figure 1:**
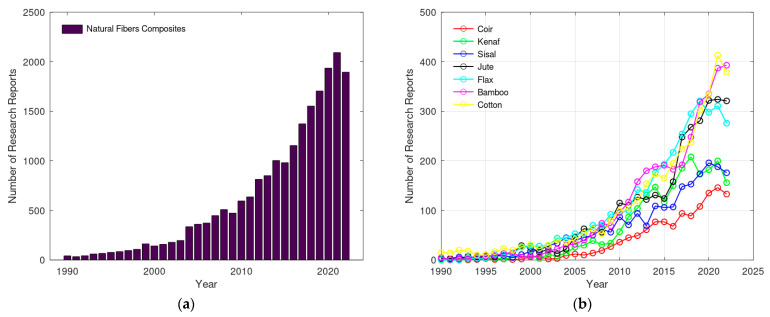
Number of studies published in the Scopus [13] database between 1990 and 2022: (**a**) composites with natural fibers and (**b**) composites by type of natural fiber reinforcement.

Since the 1990s, there has been an increased use of NLF-reinforced composites, which have been extensively studied, with several published studies presented in the Scopus database [13] as can be seen in Figure 1a. Figure 1b shows that the most recurrent fibers in these publications are cotton, sisal, jute, flax, bamboo, and coconut fibers. Together, they represented more than 92% of all scientific production in the Scopus database [13] published since 1990 in the field of natural fiber composites. In this growing universe, the jute fiber, present in the Amazon biome, occupies a prominent position in 15.3% of all bibliographic production, configuring itself as one of the most promising alternatives to synthetic fibers.

Jute fiber from the species *Corchorus capsularis* is characterized by its low weight and relatively good mechanical resistance and can naturally reach large diameters and lengths, making it an attractive option under this specific issue for structural applications [14,15,16,17]. The physical and mechanical properties of jute fiber found in the works of Tanguy et al. [18], Awais et al. [19], Ribeiro et al. [20], Ganesan et al. [21], Ahamed et al. [22], and Khalid et al. [23] are shown in Table 1.

Jute fibers have been used together with several matrices in the production of composites, as reported in the work of Khalid et al. [23], intended for numerous structural and semi-structural applications owing to their mechanical properties and the properties achieved by their composites.

Ahamed et al. [22] produced an epoxy matrix (EP) composite reinforced by chemically treated jute fibers with 46 vol%, and it reached a tensile strength of 92.74 MPa and a Young’s modulus of 5.45 GPa.

Chaturvedi et al. [24] produced three-phase hybrid composites of epoxy/jute blanket/granite particulate waste and obtained a maximum tensile strength of 35.42 MPa and a Young’s modulus of 3.64 GPa in the composites with an addition of 30 wt% granite.

Ribeiro et al. [20] manufactured composites of a polyester matrix with 25 vol% of unidirectional jute fibers, obtaining an improved performance in tensile strength of 62.11 MPa and a Young’s modulus 3.18 GPa. 

Sajin et al. [25] studied composites of a polyester matrix reinforced with 40 wt% of jute fibers with lengths between 5 and 25 mm, obtaining better results with fibers of 20 mm in length and reaching a tensile strength of 50.26 MPa and Young’s modulus of 2.3 GPa.

Zaghloul et al. [26], in evaluating the development of polyester matrix composites reinforced with natural fiber nanoparticles, concluded that resistant structural components could be manufactured using jute nanoparticles. 

Senthilrajan et al. [27], by quasi-static and dynamic mechanical tests, studied the performance of polyester/short randomly oriented jute fiber composites and concluded that in the tested conditions, jute could substitute E-glass fiber in thin-wall structural elements, highlighting the advantage of jute being cheaper and biodegradable, which reduces the cost of the products. 

In the study by Stanciu and Nastac [28], the mechanical properties of synthetic materials used in the construction of wind turbine blades were observed from samples tested for mechanical, static, and/or dynamic stresses under environmental conditions and failure criteria based on the classical laminate theory (CLT).

In the work of Mekonnen and Mamo [29] on hybrid composites with jute and bamboo fibers in various proportions, promising results were obtained for the application of NLFs in wind turbine blades.

Pickering et al. [30] showed that polyester turbine blades reinforced with NLFs and processed by resin transfer molding (RTM) are a potential substitute for glass fiber-reinforced blades, specifically recognized through the Asia 2013 Innovation Award from the JEC composites group [30].

Therefore, it is noted that composite materials reinforced with natural fibers have potential for application as wind turbine blades and may be substitutes for conventional materials, which, from an environmental point of view, would be an advantage in relation to conventional blades. Moreover, it can also be inferred that these composites have desirable mechanical properties for this type of application, presenting similar mechanical behavior to materials commonly used in the wind generation industry. 

In general, modern turbine blade materials are expected to provide high energy efficiency, low weight, low cost, sustainability, and extended durability [31]. In this context, the objective of the study is the development of composites reinforced with continuous and aligned jute fibers in the form of jute yarn and jute carded fiber, a stage before yarn in the manufacturing line, using epoxy resin as the matrix. This has not yet been explored in works of structural application with the possibility of replacing the synthetic fibers already used in the construction of blades and blade segments in the wind industry. Moreover, for the first time, the physical and mechanical properties of the composites are evaluated, as well as the influence of the orientation of the jute and carded jute yarn on the tensile and shear properties of the composites.

Low wind intensity is very common in the Amazon region of the municipality of Benevides-PA, Brazil, with coordinates 1.315456 S, 48.288359 W; making it a strong competitor for the installation of a wind turbine, as it is located far from large centers, it is difficult to access the energy networks already installed, and it is a holder of low annual wind averages of 4.71 m/s, with wind potential of 123 W/m^2^, according to the wind chart [32].

## 2. Materials and Methods

### 2.1. Materials

**Figure 2:**
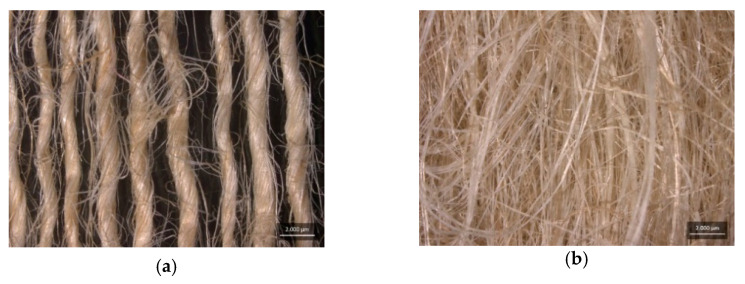
Arrangement of (**a**) jute yarn and (**b**) carded fiber.

In this study, jute fiber (*Corchorus capsularis*) was used as reinforcement for the composite in the condition of jute yarn (JY) and carded fiber (CF), supplied by Companhia Textil de Castanhal S.A (Para-Brazil), as show in Figure 2. 

The JY used in this work was characterized by obtaining its average diameter, its density, and the surface twist that forms the yarn itself. The CF was characterized in terms of its diameter and density. Epoxy resin of medium viscosity was used as the composite matrix, manufactured by Ara-chemical under the trade name Center Epoxy GY 250, and Center Aradur 63-IP, 33 wt%, was used as hardener, also supplied by Ara-chemical SA, São Paulo, Brazil.

### 2.2. Characterization of Jute Yarn (JY) and Carded Fiber (CF)

#### 2.2.1. Diameter Determination

To characterize the reinforcement fibers of the composite, a Carl Zeiss optical stereoscope, (Berlin, Germany), model Stemi 508, AXIO-CAM camera, was used to measure the diameters of 30 JY samples and 40 FC samples, where 2 measurements were taken at 90° to each other in 4 points along the length of the sample.

#### 2.2.2. Twist Angle Determination

The JY twist angle was obtained as indicated by Smail et al. [33], using a Carl Zeiss optical stereoscope, (Berlin, Germany), model Stemi 508, AXIO-CAM camera, on 30 jute yarn samples with longitudinal and transverse measurements along the length.

#### 2.2.3. Fiber Density Determination

The density of JY and FC were determined using the pycnometer technique, in a precision balance of 0.0001 g model Marte AY220. The initially dried samples were weighed and immersed in methyl alcohol (Methanol ACS CH30H-PM 32.04 Synth Factory) at 23 °C; it was then that the density was calculated and presented in g/cm^3^ according to the procedure of Ribeiro et al. [20].

### 2.3. Confection of Test Specimens

Based on the works of Maciel et al. [6] and Pires et al. [34], the epoxy matrix (EP) specimens were fabricated using a silicone mold following ASTM D 638 [35], and the JY-EP and CF-EP specimens with 30 wt% reinforcement were produced using the resin infusion process. For tensile and shear tests, the ASTM D3039 [36] and ASTM D5379 [37] standards were followed, respectively.

**Figure 3:**
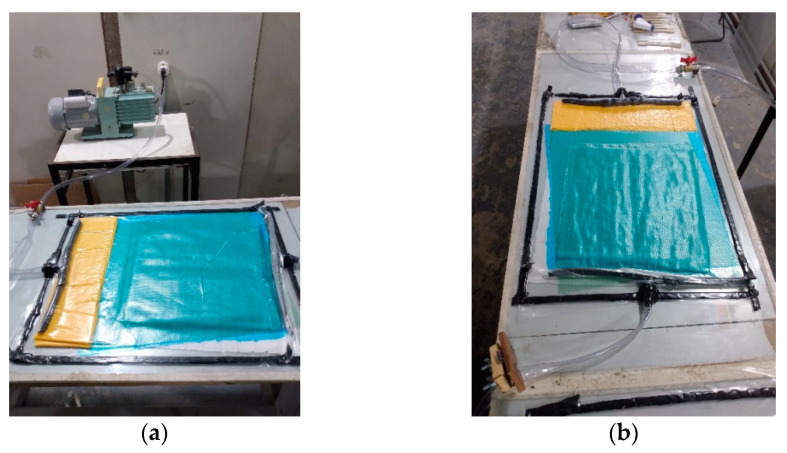
Manufacture of the composite used: (**a**) sealing tapes of the vacuum bag, hoses, and valves; (**b**) plate production process.

Figure 3 shows the production process of the plates.

Table 2 shows the compositions studied, the orientation of the reinforcement, and the number of specimens fabricated.

### 2.4. Density and Voids Determination

The density of the matrix and composites was obtained using an analytical balance with 0.0001 g precision, model Marte AY220, and a 100 mL beaker with distilled water.

The samples in EP and of JY-EP and CF-EP in discs of 50 mm of diameter were dried and immersed in distilled water according to the procedure indicated by Ribeiro et al. [20], and the density of the composites was calculated following ASTM D792 [38] and presented in g/cm^3^ using the equation

(1) $$\mathrm{sg}23{}^{\circ}=a/\left(a+w-b\right)$$

where sg23° is the density of the material at 23 °C; and a, w, and b are the dry mass of the specimen in a free air environment, the specimen mass added, and the mass of the partially immersed wire, respectively.

The voids volume (Vv) was calculated based on the classical laminate theory (CLT) through the equation

(2) $$\mathrm{Vv}=1-\rho c\left(\frac{\mathrm{wf}}{\rho f}\right.+\left.\frac{\mathrm{wm}}{\rho m}\right)$$

where ρf, ρm, and ρc are the fiber density, matrix density, and composite density, respectively; and wf and wm are the fiber mass fraction and matrix mass fraction, respectively, which were determined experimentally.

### 2.5. Mechanical Tests

#### 2.5.1. Tensile Testing

The tensile tests on the EP, JY-EP, and CF-EP were performed using universal machine model AROTEC WDW-100E (São Paulo, Brazil), with a test speed of 2 mm/min and a load cell of 5 kN.

The tests on the EP specimens with dimensions (165 × 20 × 3.89 mm) were performed according to the guidelines of ASTM D 638 [35], and the JY-EP and CF-EP with dimensions (250 × 25 × 2.5 mm) were tested according to the recommendations of ASTM D3039 [36], all instrumented with strain gauges of 120 Ohms in the longitudinal and transverse directions and with a clip-on blade strain gauge.

**Figure 4:**
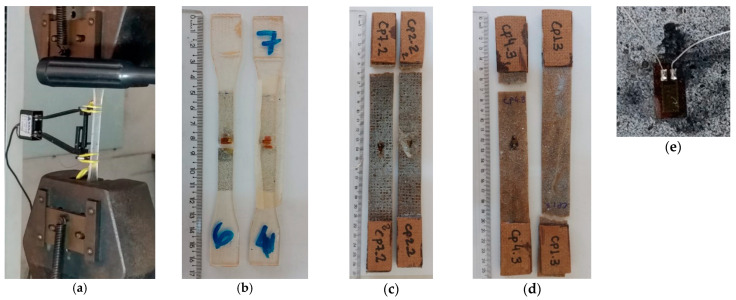
Specimens of epoxy resin and composites reinforced with jute fibers (**a**) under tensile test, (**b**) fractured after test. (**c**) JY/EP specimens, fractured under tensile testing; (**d**) CF/EP specimens, fractured under tensile testing; and (**e**) strain gauge used in the tests to obtain the results.

The Young’s modulus E_1_ of each specimen was obtained experimentally using the equation

$$E_{1}=\frac{\Delta T}{\Delta \varepsilon }$$

where ΔT and Δε are the stress variation and strain variation, respectively, measured in the elastic region of the specimen obeying Hooke’s law. The specimens of EP and of JY-EP and CF-EP are shown in Figure 4.

#### 2.5.2. Shear Testing

**Figure 5:**
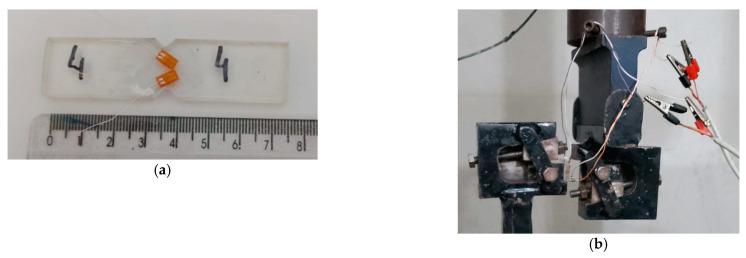
(**a**) Epoxy matrix specimen, (**b**) shear test with strain gauge used in the tests.

The Iosipescu or V-Notch shear tests were used to determine the shear strength and shear modulus, executed in a universal machine, model AROTEC WDW-100E, with a speed of 1.5 mm/min and a load cell of 5 kN. The shear test EP is shown in Figure 5.

The specimens of EP and of JY-EP and CF-EP were tested according to ASTM D5379 specifications [37]. Shear tests were instrumented with 120-Ohm strain gauges and reinforcement fibers aligned at 0° and 90°.

### 2.6. Scanning Electron Microscope (SEM)

The fracture surface of the tensile specimen was observed in a Tescan scanning electron microscope, Vega 3 model (Brno, Czech Republic) equipped with a Quorum Q150R ES metallizer (Brno, Czech Republic), 7.6 × 10^−2^ Pa vacuum chamber, and a 5 kV electron emission source.

### 2.7. Modeling

Based on the best mechanical results of the composites produced, computational modeling was carried out using the Matlab software, MathWorks 2016s version of a blade for wind turbines, using the S1210 profile to obtain the parameters of nominal power, tip rotation speed, and blade length. 

### 2.8. Statistical Analysis

Analysis of variance (ANOVA) was used to verify the possibility of significant differences between the results obtained for tensile properties and shear, adopting Tukey’s test to complement and validate the evaluation [39,40,41,42,43]. 

## 3. Results and Discussions

### 3.1. Diameter and Twist Angle Characterization 

**Figure 6:**
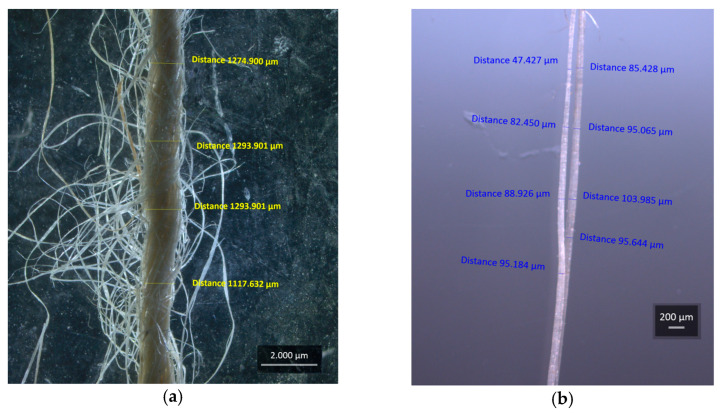
Macrograph of jute (*Corchorus capsularis*) in (**a**) the JY with diameter measurements and in (**b**) the diameter of the CF.

Figure 6 shows the average diameters of the JY and CF, obtained using stereo microscopy.

The samples of JY and CF reached average diameters of 1.07 mm and 0.0867 mm, respectively, measured at four points along the samples, shown in Figure 6a for JY and in Figure 6b for CF, which were closer to the average diameters obtained.

**Figure 7:**
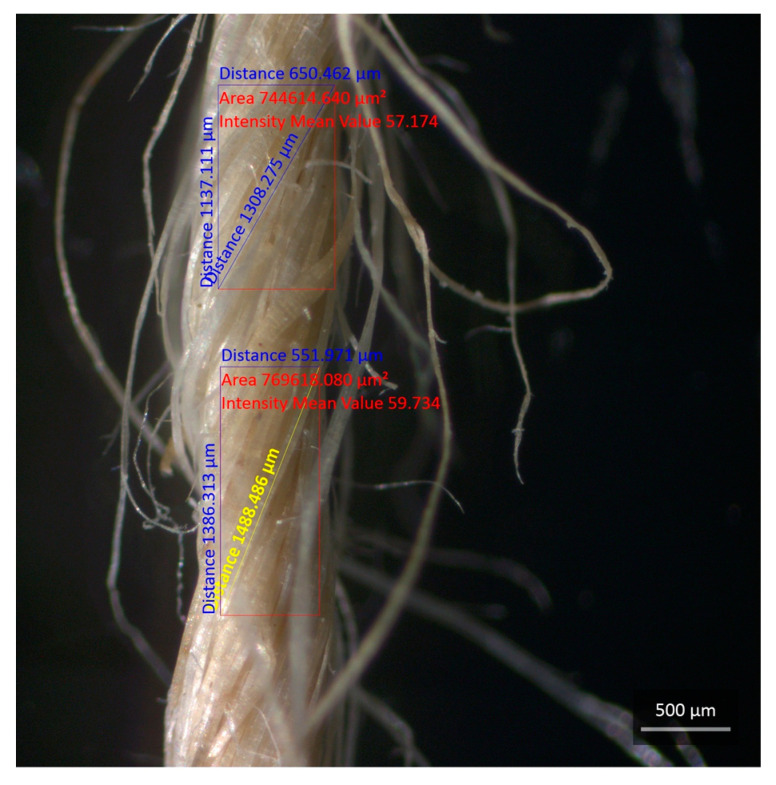
Macrograph of JY to obtain the twist angle of the yarn, using trigonometry.

Figure 7 shows the way of capturing the JY torsion angle through the stereo microscope and calculation by trigonometry.

The results found an average torsion angle in JY of 18.5°, which reaffirms it for a structural application, equating to the results of Khan et al. [43] and Jesus et al. [44]. The average values presented are close to the results of Shi et al. [45].

### 3.2. Results of Applied Tests

#### 3.2.1. Physical Properties of Fibers

The results found for the density of JY were 1.029 g/cm^3^ and 1.17 g/cm^3^ for CF, obtained through the pycnometer technique. 

#### 3.2.2. Physical Properties of Composites

**Figure 8:**
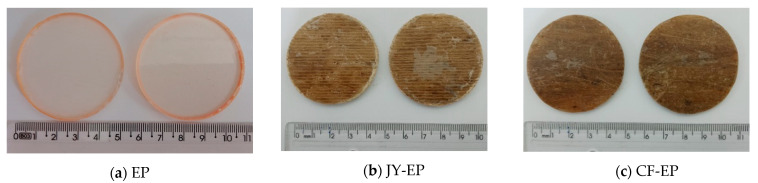
Composite discs: manufactured by resin infusion.

Figure 8 shows the EP, JY-EP, and CF-EP materials on 50 mm diameter discs taken from plates produced by resin infusion for the density tests.

Table 3 presents the results of the density and void volume tests of the matrix and composites.

It is observed that the density of the EP, JY-EP, and CF-EP reached 1.16, 1.25, and 1.24 g/cm^3^, respectively. The void fractions (Vv) obtained from the tests on the EP and the JY-EP and CF-EP were 4.4%, 5.0%, and 6.5%, respectively. The results of this work were close to the results obtained with jute/coir/polyester (J-C-PE) by Ganesan et al. [21], suggesting that the composite manufacturing process promoted an increase in density when compared to the matrix.

#### 3.2.3. Mechanical Properties of Composites in Tensile

Table 4 shows the average results of the mechanical properties of the EP and the JY-EP and CF-EP composites in the tensile test. In this table, the reinforcement alignment and mass fraction of the studied composites are indicated.

Analyzing the results in Table 4, in tensile strength (MPa), it is observed that that of JY-EP 0° is 15% lower and that of CF-EP 0° is 14.4% higher than that of the EP. For the results in Young’s modulus (GPa), it is observed that that of the JY-EP 0° is 173.3% higher and that of the CF-EP 0° is 15.5% higher than that of the EP. The tensile strength, Young’s modulus, and total strain were found in the EP as 41.434 MPa, 3.608 GPa, and 0.0266 mm/mm; in the composite JY-EP 0° as 47.398 MPa, 9.860 GPa, and 0.0169 mm/mm; and in the composite CF-EP 0° as 35.307 MPa, 4.169 GPa, and 0.0088 mm/mm, respectively. Regarding the total strain (mm/mm), it is observed that that of JY-EP 0° is 36% lower and that of CF-EP 0° is 67% lower than that of the EP.

**Figure 9:**
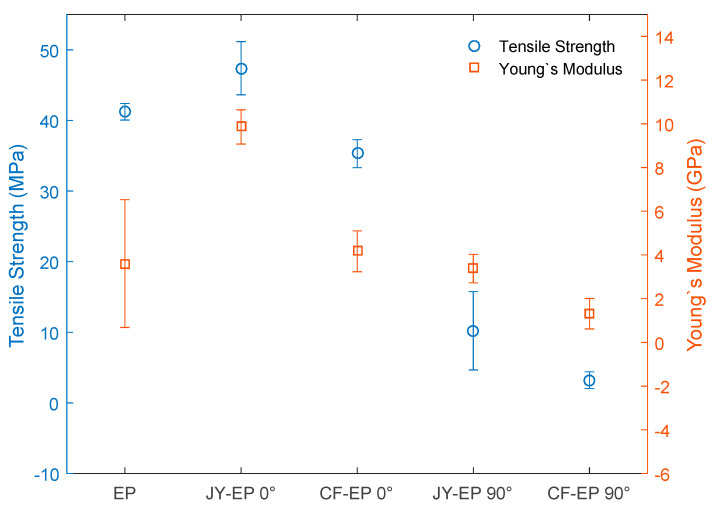
Mechanical properties of composites.

The comparative strain and Young’s modulus results are presented in the graph in Figure 9.

Comparing the results of the tensile tests in Figure 9, one finds that the maximum tensile strengths (MPa) of JY-EP 0° and CF-EP 0° are lower and higher, respectively, than the results of the EP. Moreover, the results of the composites’ Young’s moduli (GPa) are higher compared with those of the EP, and those of the total strain (mm/mm) are superior in comparison with the results of the EP; while JY-EP 90° and CF-EP 90° disclose a lower tensile strength (MPa), Young’s modulus (Gpa), and total strain (mm/mm) than the results of the EP. The results found show that the addition of fibers aligned at 0° promote effective reinforcement of the matrix, when requested in traction, and suggest that higher levels of JY could be added to the matrix, when requested in traction. The same pattern was not observed when JY was added at 90° and CF at 0 and 90°, which shows that the type and orientation of the wire influences the mechanical properties of the composites. As for the Young’s modulus, it is observed that the JY-EP 0° promoted an increase in the stiffness of the composite; however, this pattern was not observed for the other composites studied, suggesting that the type of wire and the orientation directly influence this property. 

Shah et al. [46] presented results of a composite with an epoxy matrix using flax and sisal as reinforcement fibers, achieving properties of a maximum tensile strength of 39.22 MPa, a Young’s modulus of 2.37 GPa, and a density of 1.158 g/cm^3^.

The work of Kalagi et al. [47] investigated an epoxy matrix composite with jute and coconut fibers and obtained a tensile strength of 37.5 MPa, a Young’s modulus of 1.5 GPa, and a density of 1.2214 g/cm^3^.

The work of Lau et al. [48] presented comparative results between epoxy matrix composites with hemp fibers and an epoxy matrix with jute fibers, obtaining results of a maximum tensile strength and Young’s modulus of 75 MPa and 3.33 GPa for hemp and 58 MPa and 4 GPa for jute, respectively.

The present results, in mass fractions of the composites with jute and carded jute fiber of JY-EP 30 wt% and CF-EP 30 wt%, were approximate if compared with the work of Shah et al. [46] using flax and glass fibers 26.9 wt% and 30 wt%, respectively, as in the work of Lau et al. [48] using 40 wt% hemp fibers. On the other hand, the density of the JY-EP and CF-EP composites was approximately 23% lower than those of the glass fiber composite studied in the work of Shah et al. [46] with values of 1.29 g/cm^3^ and 1.64 g/cm^3^ for flax and glass fibers, respectively. Comparing the maximum tensile strength of the JY-EP and CF-EP composites, 47.4 MPa and 35.3 MPa, respectively, the results are 17.2% higher than those of the composites with glass fibers from the study of Shah et al. [46], for example. The Young’s modulus achieved in this study is equivalent to the results presented in the literature.

**Figure 10:**
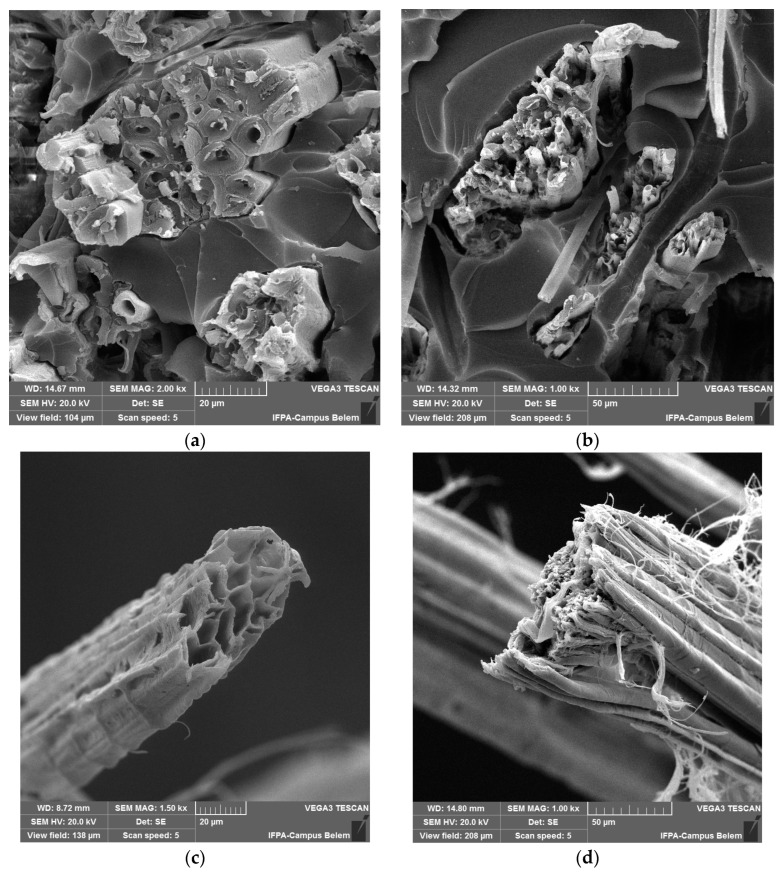
Fracture surface showing the failure modes present in composites: (**a**) fiber and matrix, (**b**) river marks, (**c**) jute fiber and aveolar section, (**d**) Carded fiber and align fibers.

Figure 10 shows SEM images, which show the fracture surface of the composites and the failure modes that occurred in the tensile test.

The fracture surface of the tested composites shows the dominant failure mode in the fiber rupture in Figure 10a,b. Furthermore, the occurrence of a smaller degree of fiber/matrix detachment is revealed in Figure 10a and river marks are shown in Figure 10b. Moreover, Figure 10c and Figure 10d show a honeycomb structure on the face with a rough fiber surface and fiber alignment forming a bundle, respectively.

#### 3.2.4. Mechanical Properties of Composites in Shear

**Figure 11:**
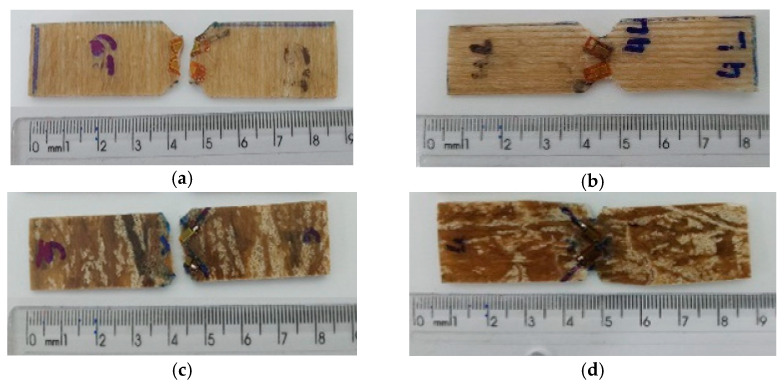
Specimens tested in shear according to ASTM D5379 standard: (**a**) jute yarn 0° (JY-EP 0), (**b**) jute yarn 90° (JY-EP 90), (**c**) fiber 90° carded jute (CF-EP 90), and (**d**) 0° carded jute fiber (CF-EP 0).

Figure 11 shows the specimens of the composites JY-EP and CF-EP after shear tests.

The specimens that were shear-tested broke by crushing and notch fracturing, as shown in Figure 11a–d. The results disclosed rupture within the acceptable standards indicated by ASTM D 5379 [37] and that the composites achieved mechanical strength consistent with the shear tests.

Table 5 shows the numerical average of the shear test results of the EP as well as the JY-EP 0°, CF-EP 0°, JY-EP 90°, and CF-EP 90° tested. It also shows the orientation of the reinforcing fiber and the studied compositions.

Analyzing the shear test results in Table 5 in terms of the maximum shear strength (MPa), it is observed that the JY-EP 0° result is 3.17% lower and the CF-EP 0° result is 19.4% lower than that of the EP. For the results of the shear modulus (GPa), the JY-EP 0° result is 67.7% lower and the CF-EP 0° result is 125% higher than that of the EP, respectively. Regarding the results of shear strain (mm/mm) it is observed that the JY-EP 0° result is 39.8% and the CF-EP 0° result is 79.2% higher than that of the EP.

**Figure 12:**
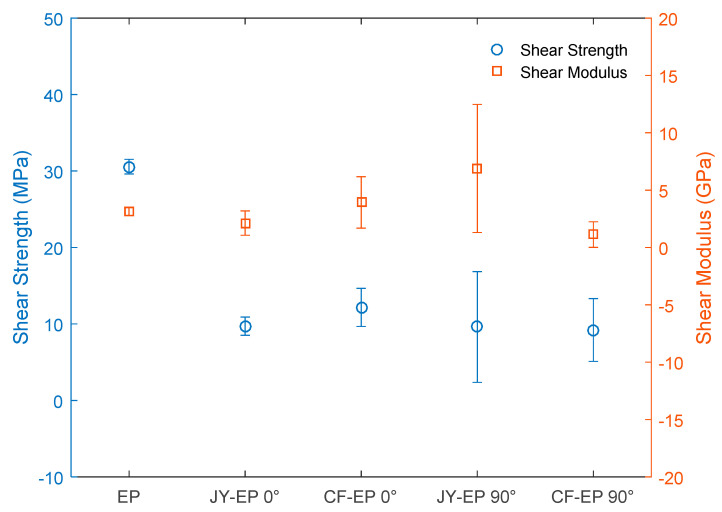
Comparative results of shear properties.

Figure 12 shows the graph of the comparative average results of the shear strength and shear modulus of the EP and JY-EP 0° as well as CF-EP 0°, JY-EP 90°, and CF-EP 90°. The calculated standard deviation of the results obtained in each composition of the material studied is also shown.

Comparing the results in the shear tests between the EP and the JY-EP 0° and CF-EP 0° composites, the composites’ values of shear strength are lower than that of the EP, while the shear modulus is lower and higher than that of the EP, respectively. In addition, shear strain (mm/mm) values higher than that of the EP, respectively, were achieved.

Comparing the obtained shear strength and shear strain results of composites JY-EP 90° and CF-EP 90°, they were lower than that of the EP, while their shear moduli were higher and lower values, respectively. 

The results obtained showed that JY and CF did not reinforce the matrix when requested in shear, and the orientation of the wires did not influence this property either, which shows that for this type of mechanical request, the added volumetric fraction showed whether it was insufficient and what other quantities could be tested. With regard to the shear modulus, within a statistical error, no significant difference was observed. Despite this result, it is believed that when producing a laminated composite, the results in shear can be effective when comparing the matrix with these composites.

### 3.3. Statistical Analysis

#### 3.3.1. Statistical Analysis in Tensile

Table 6 shows the results of the ANOVA statistical analysis on the tensile strength, Young’s modulus, and total strain for the tensile test of the EP and the JY-EP 0°, CF-EP 0°, JY-EP 90°, and CF-EP 90° composites. 

As observed in the properties of tensile strength, Young’s modulus, and total strain, the values of F, calculated as (353.88), (26.14), and (4.3263), respectively, are higher than the value of F critical (2.76). Thus, the null hypothesis is rejected with significant statistical differences between the properties of different types at a 95% confidence level. For confirmation of the ANOVA results, the Tukey test was performed for tensile mechanical properties.

Table 7 presents the results obtained in the Tukey test for the confirmation of the ANOVA results.

The number of the minimum significant difference (m.s.d.) indicates which treatment presents a difference in its mean values. When the mean values of the groups compared two by two are greater than the m.s.d. value, it is considered that there is a difference between the average values evaluated. The m.s.d for the maximum strength was calculated as 1.287, the m.s.d for Young’s modulus was calculated as 0.752, and the m.s.d for total strain was 0.00711; therefore, it is observed that the tensile strengths of the composites JY-EP 0°, CF-EP 0°, JY-EP 90°, and CF-EP 90° are different from each other, with a confidence level of 95%.

#### 3.3.2. Statistical Analysis in Shear

Table 8 shows the results of the ANOVA statistical analysis for the shear test concerning the shear strength, shear modulus, and shear strain in the EP and the JY-EP 0°, CF-EP 0°, JY-EP 90°, and CF-EP 90° composites.

Based on the results of ANOVA, it can be observed that for the shear strength, the F calculated (1.356) is lower than the F critical value (3.48), and for the shear modulus, the F calculated (0.65) is lower than the F critical value (3.48). Therefore, the hypothesis that the average values are equal is accepted, with a confidence level of 95%. On the other hand, the shear strain presented the F calculated (17.4266) higher than the value of the F critical (3.48). Therefore, the hypothesis that the average values of shear strain are equal, with a confidence level of 95%, was rejected, and Tukey test was applied to discriminate which group showed significant differences for this property. 

Table 9 presents the Tukey test results for the shear strain obtained from the shear test.

The m.s.d for the shear strain values was calculated as 0.00361. Thereby, 0EP and JY-EP 0° are different from all compositions, CF-EP 0° is also different from JY-EP 90° and from CF-EP 90°, and finally, JY-EP 90° is also different from CF-EP 90°. Thus, it can be seen that fiber orientation influences the shear strain property. 

### 3.4. Computational Modeling of Blades for the S1210 Profile and Environmental Considerations

The prediction of the aerodynamic model for the S1210 profile, shown in Table 10, showed that for a blade with a length of 1.64 m, the rated power was 62 kW, showing that smaller blades are capable of generating energy, even in regions of low wind intensity. Kalagi, Patil, and Nayak [47] showed that a blade with a length of 3.5 m had a rated power of 100 kW, suggesting that the blade produced in the present study can be manufactured in larger dimensions, thus increasing its power generation capacity. 

Table 10 presents the characteristics of the horizontal axis wind blade for low-intensity winds, where the results of power generation and blade tip speed reached 62 kW and 24.23 m/s, respectively, being 38% lower and 49% higher in power generation and blade tip speed compared to that reported by Kalagi, Patil, and Nayak [47]. 

Wind turbine blades for high-intensity winds generally have a length ranging from 40 to 70 m and are capable of converting the kinetic energy of the air into movement energy in the turbine of the wind turbines [49]. These blades are manufactured from polymeric matrices of epoxy or polyester, reinforced with glass or carbon fibers aiming at lower weight and better mechanical performance, making these blades present a high Young’s modulus and high static and dynamic loads [49,50,51]. Blades for wind turbines for application in low-intensity regions also need the same structural and mechanical requirements but smaller dimensions due to lower wind intensity. Thus, the projection of blades proposed in this work suggests that epoxy matrix composites reinforced with jute yarn oriented at 0° would be alternatives for this type of application. Furthermore, the material used to manufacture this material is more sustainable than conventionally used materials. In this context, in addition to the shape parameters of the blades and the energy generation capacity, another important factor is the correct disposal of the materials that constitute it after the end of its use in work. The blades proposed in this work, in addition to being mechanically viable, have advantages over those currently used, as they are partly composed of natural materials and are biodegradable. Furthermore, the composite can be mechanically recycled in order to produce particulates that can be used as raw material for the manufacture of other materials such as construction materials or be reincorporated in the production of blades. It is also noteworthy that in addition to the possibility of recycling, small-sized blades can be advantageous in terms of lower noise emissions, which make them attractive for application in urban areas and with low wind intensity. 

## 4. Summary and Conclusions

In this work, the jute fiber grown in the Brazilian Amazon region and processed into the forms of yarn and carded fiber was used as reinforcement in composites with epoxy matrices in small wind blades.

The main properties found in the studied materials of JY-EP and CF-EP reached average densities of 1.17 and 1.029 g/cm^3^, respectively, which resulted in relatively lower densities in plant fiber, when compared to the current literature.

In the tensile test for JY-EP and CF-EP, the tensile strength results were 47.4 and 35.31 MPa, the Young’s modulus results were 9.86 and 4.17 GPa, and the total strain results were 0.0169 and 0.0088 mm/mm, respectively. The properties found in the shear test in shear strength were 9.169 and 12.712 MPa, and those in the shear modulus were 2.129 and 3.93 GPa, respectively, proving that composite materials such as JY-EP 30 wt% and CF-EP 30 wt% compared to fibers such as flax, sisal, hemp, and coconut are good options for use in laminated structures applied to small wind blades.

The images obtained by SEM showed that there was no pull-out of the fiber in relation to the matrix, which is one of the main defects in composite materials with the rupture occurring practically at the same time, confirming the potential of the manufacturing technique.

In this work, the use of jute fibers reinforcing EP was evaluated for the construction of the blades of a small-scale wind turbine with application in regions of low wind speed. This is a renewable option for reinforcement material, alone or in consortium with synthetic fibers, reducing the environmental impact of the growing volume of parts and components under disposal from the wind power industry.

## Figures and Tables

**Table 1:** Jute fiber properties.

Density (g/cm^3^)	Diameter (μm)	Strain Failure (%)	Tensile Strength (MPa)	Young’s Modulus (Gpa)	Reference
-	40.5	2.01	277	15.6	[18]
1.3–1.5	-	1.5–1.8	200–800	20–55	[19]
1.482	78	1.7–4.1	380.87	17.95	[20]
1.5	90–115	1.16–1.80	393–773	19.0–26.5	[21]
1.48	45	1.1	270	33	[22]
1.4	-	1.5–1.7	390–770	20–30	[23]

**Table 2:** Characteristics of the samples used.

Composition	Nomenclature	Orientation	Quantity Matrix	Quantity Fibers	Number of Samples for Tensile Testing	Number of Samples for Shear Test
		(°)	(wt%)	(wt%)		
	Epoxy Resin	-	100	0	6	3
JY-EP 0°	Jute Yarn-Epoxy Resin	0	70	30	6	3
JY-EP 90°	Jute Yarn-Epoxy Resin	90	70	30	6	3
CF-EP 0°	Carded Fiber-Epoxy Resin	0	70	30	6	3
CF-EP 90°	Carded Fiber-Epoxy Resin	90	70	30	6	3

**Table 3:** Results of physical properties of composites.

Composite 30 wt.%	Density (g/cm^3^)	Voids Fractions (%)	
EP	1.16	4.4	This work
JY-EP	1.25	5.0	
CF-EP	1.24	6.5	
J-C-PE *	1.195	4.4	[21]

* J-C-PE: Jute/coir/polyester.

**Table 4:** Tensile test results of composites.

Composite	Orientation	Fiber Volume Fraction	Tensile Strength	Young’s Modulus	Total Strain
	(°)	(%)	(MPa)	(GPa)	(mm/mm)
EP	-	0	41.434 ± 1.182	3.608 ± 2.924	0.0266 ± 0.018
JY-EP 0°	0°	30	47.398 ± 3.771	9.860 ± 0.784	0.0169 ± 0.012
CF-EP 0°	0°	30	35.307 ± 5.546	4.169 ± 0.650	0.0088 ± 0.001
JY-EP 90°	90°	30	10.215 ± 1.982	3.375 ± 0.935	0.0351 ± 0.023
CF-EP 90°	90°	30	3.535 ± 0.919	1.423 ± 0.695	0.0041 ± 0.002

**Table 5:** Composite shear test results.

Composite	Orientation	Fiber Volume Fraction	Shear Strength	Shear Modulus	Shear Strain
	(°)	(%)	(MPa)	(GPa)	(mm/mm)
0EP	-	0	30.580 ± 0.9569	3.142 ± 0.328	0.00530 ± 0.0043
JY-EP 0°	0°	30	9.712 ± 1.1944	2.129 ± 1.061	0.00355 ± 0.0005
CF-EP 0°	0°	30	12.169 ± 2.4899	3.930 ± 2.2462	0.00198 ± 0.0035
JY-EP 90°	90°	30	9.602 ± 7.241	6.897 ± 5.588	0.002637 ± 0.0021
CF-EP 90°	90°	30	9.204 ± 4.1084	1.130 ± 1.1165	0.005753 ± 0.0027

**Table 6:** ANOVA for tensile strength of the composites.

Maximum Strength (MPa)						
Source	Sum of Squares	Degrees of Freedom	Mean of Squares	F (Calculated)	F Critical	*p*-Value
Between the groups	9453.70	4	2363.43	353.88	2.76	1.0 × 10^−21^
Inside the group	166.96	25	6.68			
Total	9620.66	29				
**Young’s Modulus (GPa)**						
**Source**	**Sum of Squares**	**Degrees of Freedom**	**Mean of** **Squares**	**F** **(Calculated)**	**F Critical**	***p*** **-Value**
Between the groups	238.01	4	59.50	26.14	2.76	1.0 × 10^−8^
Inside the group	56.90	25	2.28			
Total	294.91	29				
**Total Strain (mm/mm)**						
**Source**	**Sum of Squares**	**Degrees of Freedom**	**Mean of** **Squares**	**F** **(Calculated)**	**F Critical**	***p*** **-Value**
Between the groups	0.0035	4	8.83 × 10^−4^	4.3263	2.76	8.59 × 10^−3^
Inside the group	0.0051	25	2.04 × 10^−4^			
Total	0.0086	29				

**Table 7:** Tukey test for tensile test of composites.

Maximum Strength (MPa) (m.s.d = 1.287)					
	0EP	JY-EP 0°	CF-EP 0°	JY-EP 90°	CF-EP 90°
0EP	-	0.004	0.23	0.0001	0.0001
JY-EP 0°	**5.66**	-	0.0001	0.0001	0.0001
CF-EP 0°	**3.05**	**8.71**	-	0.0001	0.0001
JY-EP 90°	**29.59**	**35.24**	**26.53**	-	0.0014
CF-EP 90°	**35.92**	**41.58**	**32.87**	**6.33**	-
**Young’s Modulus (GPa)** **(m.s.d. = 0.752)**					
	**0EP**	**JY-EP 0°**	**CF-EP 0°**	**JY-EP 90°**	**CF-EP 90°**
0EP	-	0.0001	**0.98**	**0.96**	**0.050**
JY-EP 0°	**9.54**	-	0.0001	0.0001	0.0001
CF-EP 0°	**0.76**	**8.79**	-	0.73	0.015
JY-EP 90°	**0.98**	**10.53**	**1.74**	-	0.20
CF-EP 90°	**4.16**	**13.70**	**4.91**	**3.17**	-
**Total Strain (mm/mm)** **(m.s.d. = 0.00711)**					
	**0EP**	**JY-EP 0°**	**CF-EP 0°**	**JY-EP 90°**	**CF-EP 90°**
0EP	-	**0.93**	**0.45**	**0.62**	**0.17**
JY-EP 0°	**1.11**	-	**0.89**	**0.21**	**0.54**
CF-EP 0°	**2.40**	**1.29**	-	**0.034**	**0.97**
JY-EP 90°	**2.01**	**3.12**	**4.41**	-	**0.008**
CF-EP 90°	**3.30**	**2.19**	**0.90**	**5.31**	-

**Table 8:** ANOVA for composites’ shear test.

Shear Strength (MPa)						
Source	Sum of Squares	Degrees of Freedom	Mean of Squares	F (Calculated)	F Critical	*p*-Value
Between the groups	3.14 × 10^−5^	4	7.851 × 10^−6^	1.356	3.48	3.159 × 10^−1^
Inside the group	5.788 × 10^−5^	10	5.788 × 10^−6^	-	-	-
Total	8.928 × 10^−5^	14	-	-	-	-
**Shear Modulus (GPa)**						
**Source**	**Sum of Squares**	**Degrees of Freedom**	**Mean of** **Squares**	**F** **(Calculated)**	**F Critical**	***p*-Value**
Between the groups	157.40	4	39.35	0.65	3.48	6.36 × 10^−1^
Inside the group	600.51	10	60.05	-	-	-
Total	757.91	14	-	-	-	-
**Shear Strain (mm/mm)**						
**Source**	**Sum of Squares**	**Degrees of Freedom**	**Mean of** **Squares**	**F** **(Calculated)**	**F Critical**	***p*-Value**
Between the groups	5.96 × 10^−4^	4	1.49 × 10^−4^	17.4266	3.48	1.67 × 10^−4^
Inside the group	8.55 × 10^−5^	10	8.55 × 10^−6^	-	-	-
Total	0.000682	14	-	-	-	-

**Table 9:** Tukey test for composite shear test.

Shear Strain (mm/mm) (m.s.d. = 0.00361)					
	0EP	JY-EP 0°	CF-EP 0°	JY-EP 90°	CF-EP 90°
0EP	-	**0.9444**	0.0008718	**0.7963**	**0.9996**
JY-EP 0°	**1.033**	-	0.0003493	**0.9947**	**0.8822**
CF-EP 0°	**8.598**	**9.631**	-	0.0002215	0.00112
JY-EP 90°	**1.575**	**0.5419**	**10.17**	-	**0.6946**
CF-EP 90°	**0.2707**	**1.303**	**8.327**	**1.845**	-

**Table 10:** Data obtained by computational modeling for the S1210 profile for application in regions with low-intensity winds.

Composite	Wind Power	Rated Power	Tip Speed Rotation	Length Blade	
	W/m^3^	kW	m/s	m	
Jute/Epoxy	134.43	62	24.23	1.64	This work
E-Glass/Flex/Epoxy	--	100	11.90	3.50	[47]

## Data Availability

Data sharing not applicable.
